# Transcutaneous Spinal Cord Stimulation Facilitates Respiratory Functional Performance in Patients with Post-Acute COVID-19

**DOI:** 10.3390/life13071563

**Published:** 2023-07-14

**Authors:** Alexander Ovechkin, Tatiana Moshonkina, Natalia Shandybina, Vsevolod Lyakhovetskii, Ruslan Gorodnichev, Sergey Moiseev, Ricardo Siu, Yury Gerasimenko

**Affiliations:** 1 Kentucky Spinal Cord Injury Research Center, University of Louisville, Louisville, KY 40202, USA; ricardo.siu@louisville.edu (R.S.); yury.gerasimenko@louisville.edu (Y.G.); 2 Department of Neurological Surgery, University of Louisville, Louisville, KY 40202, USA; 3 Department of Physiology, University of Louisville, Louisville, KY 40202, USA; 4 Pavlov Institute of Physiology, Russian Academy of Sciences, 199034 St. Petersburg, Russia; moshonkina@infran.ru (T.M.); ellippss@gmail.com (N.S.); lyakhovetskiiva@infran.ru (V.L.); 5 Velikie Luki State Academy of Physical Education and Sports, 182100 Velikie Luki, Russia; gorodnichev@vlgafc.ru (R.G.); siranovl@yandex.ru (S.M.)

**Keywords:** neuromodulation, spinal cord stimulation, respiration, rehabilitation, COVID-19

## Abstract

Background: A growing number of studies have reported Coronavirus disease (COVID-19) related to both respiratory and central nervous system dysfunctions. This study evaluates the neuromodulatory effects of spinal cord transcutaneous stimulation (scTS) on the respiratory functional state in healthy controls and patients with post-COVID-19 respiratory deficits as a step toward the development of a rehabilitation strategy for these patients. Methods: In this before-after, interventional, case–controlled clinical study, ten individuals with post-acute COVID-19 respiratory deficits and eight healthy controls received a single twenty-minute-long session of modulated monophasic scTS delivered over the T5 and T10 spinal cord segments. Forced vital capacity (FVC), peak forced inspiratory flow (PIF), peak expiratory flow (PEF), time-to-peak of inspiratory flow (tPIF), and time-to-peak of expiratory flow (tPEF), as indirect measures of spinal motor network activity, were assessed before and after the intervention. Results: In the COVID-19 group, the scTS intervention led to significantly increased PIF (*p* = 0.040) and PEF (*p* = 0.049) in association with significantly decreased tPIF (*p* = 0.035) and tPEF (*p* = 0.013). In the control group, the exposure to scTS also resulted in significantly increased PIF (*p* = 0.010) and significantly decreased tPIF (*p* = 0.031). Unlike the results in the COVID-19 group, the control group had significantly decreased PEF (*p* = 0.028) associated with significantly increased tPEF (*p* = 0.036). There were no changes for FVC after scTS in both groups (*p* = 0.67 and *p* = 0.503). Conclusions: In post-COVID-19 patients, scTS facilitates excitation of both inspiratory and expiratory spinal neural networks leading to an immediate improvement of respiratory functional performance. This neuromodulation approach could be utilized in rehabilitation programs for patients with COVID-19 respiratory deficits.

## 1. Introduction

Management of pulmonary dysfunction is one of the most prominent problems in medical practice [1]. A global pandemic of the novel respiratory infection Coronavirus disease (COVID-19) has contributed to the complexity of this problem tremendously [2]. Although the acute phase is the most severe, the long-term consequences of this disease can persist and need to be addressed [3,4]. Most patients who developed COVID-19-induced pneumonia have bilateral lung lesions, respiratory failure, and/or acute respiratory distress syndrome (ARDS) [5] leading to significant changes in lung function, mostly restrictive, which can persist after recovery and are associated with an increased risk of life-threatening comorbidities [6,7]. Therefore, the development of rehabilitation methods to maintain adequate respiratory function are critical in this population [8].

Currently, there are no effective therapeutic strategies to improve respiratory motor function after COVID-19 that are accepted as a standard of care. A fundamental reason for this is that the pathophysiological mechanisms of respiratory motor dysfunction after COVID-19 are not known. Although the primary pathogenic target is the respiratory system, a growing number of studies have reported central nervous system manifestations of COVID-19 [9,10,11,12,13,14]. Increasing reports have shown that COVID-19 infection affecting central and peripheral nervous systems by direct or indirect damage of neurons and respiratory neural networks may lead to long-term respiratory deficits [15]. Respiratory neuroplasticity, defined as a persistent morphological and functional change in neural control based on behavioral experience, is critically dependent on the establishment of necessary preconditions, the stimulus paradigm, and a balance of complementary neuromodulation through hypoxia, hypercapnia, exercise, stress, and/or other factors [16,17,18]. These speculations suggest that neuromodulation through spinal cord stimulation has potential for respiratory rehabilitation in this patient population. Recently, we reported that non-invasive spinal cord transcutaneous stimulation (scTS) may be a viable neuromodulatory approach to affect locomotor behavior [19] and cardiovascular function [20]. The results of these studies, as well as our previous observations [21,22,23] and the work of others [24], led us to the idea that the activity of respiratory neural networks affected by COVID-19 can be neuromodulated by scTS, with the goal of utilizing this technique for respiratory rehabilitation. We hypothesized that scTS targeting spinal networks anatomically associated with innervation of the accessory respiratory muscles results in an acute increase in respiratory functional effectiveness. Here, for the first time, we propose that scTS configured for the activation of respiratory neuronal networks will result in improved respiratory functional performance in patients with post-acute COVID-19 respiratory deficits.

## 2. Materials and Methods

### 2.1. Study Design and Participants

The study was approved by the Ethics Committee of the Pavlov’s Institute of Physiology, St. Petersburg, Russian Federation (protocol #20-02 dated 18 December 2020) and was conducted in accordance with the requirements of the Ministry of Science and Higher Education of the Russian Federation “On the activities of organizations subordinate to the Ministry of Science and Higher Education of the Russian Federation in the conditions of preventing the spread of the COVID-19 infection in the territory of the Russian Federation” (order #692 dated 28 May 2020).

Data collection occurred between December 2020 and March 2022. Data sets were collected in a physiological laboratory environment during a single visit for each participant. The outcome measures were assessed prior to the intervention (“pre-intervention” time point) and 15 min after the 20-min exposure (“post-intervention” time point) to the simultaneous two-channel scTS applied over the T5 and T10 spinal cord segments. Eight healthy controls (all males, 24 ± 4 years of age, BMI 22 ± 2 kg/m^2^) and ten post-acute COVID-19 participants were recruited. Five female and five male COVID-19 participants 55 ± 13 years of age previously hospitalized with severe COVID-19 in association with pneumonia were recruited after 59 ± 48 days post-diagnosis (Table 1). Prior to entering this study, all participants were PCR-tested negative for COVID-19. The following inclusion criteria were applied: at least 21 years of age; post-acute COVID-19 syndrome; no ventilatory dependence; respiratory functional deficit defined as a decrease in predicted FVC values at least 20%; no tobacco or drug use; and no cardiovascular or respiratory conditions unrelated to COVID-19. All individuals reported episodes of shortness of breath and fatigue at the time of the study. Besides experimental procedures, all participants maintained their normal daily routines. No participants withdrew from the study.

### 2.2. Research Procedures

**Figure 1:**
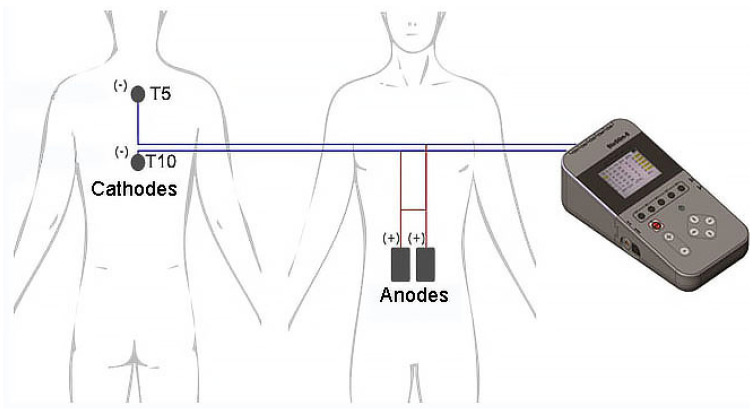
Placement of the spinal cord transcutaneous stimulation electrodes. Cathodes (−) are located on the skin between Th3-Th4 and Th8-Th9 spinous processes corresponding to T5 and T10 spinal cord segments, respectively. The anodes (+) are placed over the abdomen and centered horizontally at the umbilical level.

Multi-site scTS was delivered by a Biostim-5 device (Cosyma Inc., Denver, CO, USA) over the midline between the third and fourth and between the eighth and nineth thoracic spinous processes (Th3-Th4 and Th8-Th9) corresponding to the T5 and T10 spinal cord segments via self-adhesive electrodes (cathodes) with a diameter of 32 mm (ValueTrode, Axelgaard Manufacturing Co., LTD, Fallbrook, CA, USA) [24]. Two 5 × 9 cm interconnected self-adhesive rectangular electrodes (ValueTrode, Axelgaard Manufacturing Co., LTD, Fallbrook, CA, USA) served as anodes and were placed bilaterally along the rectus abdominus muscles centered at the umbilical level (Figure 1). Stimulation consisted of 5 kHz-modulated, monophasic, 1-ms pulses delivered with a frequency of 30 Hz to activate dorsal roots as reported previously [25], providing afferent input to the spinal cord segments involved in the activation of the accessory respiratory muscles. During the stimulation intensity mapping phase, starting at 10 mA, the current was gradually increased up to 40.1 ± 11.9 mA at Th3/Th4 and up to 40.3 ± 13.7 mA at Th8/Th9 until twitching of the intercostal or abdominal muscles was noted, indicating the motor threshold. For interventional stimulation, these values were decreased -10 mA to the sub-motor threshold levels. Brachial arterial blood pressure (BP), heart rate (HR), oxygen saturation, and pain sensation using a visual analogue scale of pain (VASp) were monitored 20 min before, during, and 20 min after the intervention.

### 2.3. Outcome Measures and Statistical Analysis

Respiratory function outcome measures: forced vital capacity (FVC, L), peak forced inspiratory flow (PIF, L/min), peak expiratory flow (PEF, L/min), time-to-peak of inspiratory flow (tPIF, sec), and time-to-peak of expiratory flow (tPEF, sec) were obtained from flow-volume curves recorded in the seated position during standard spirometry using a Powerlab 16/35 data acquisition system with the Human Respiratory Kit (AD Instruments, Denver, CO, USA) [26]. Peak flow outcomes and time points to achieve these levels, reflecting the effectiveness of accessory respiratory muscular engagement and recruitment rate, were used as indirect measures of respiratory neuromuscular activity [27].

Data values were analyzed in R (R Foundation for Statistical Computing, Vienna, Austria) and represented by descriptive statistics (mean ± standard deviation). Pre- to post-intervention changes for each outcome measure were tested for normality using the Kolmogorov–Smirnov and the Shapiro–Wilk tests. A paired *t*-test and signed rank test were used to assess normally and non-normally distributed outcomes, respectively. The statistical significance threshold was set to α = 0.05.

## 3. Results

**Figure 2:**
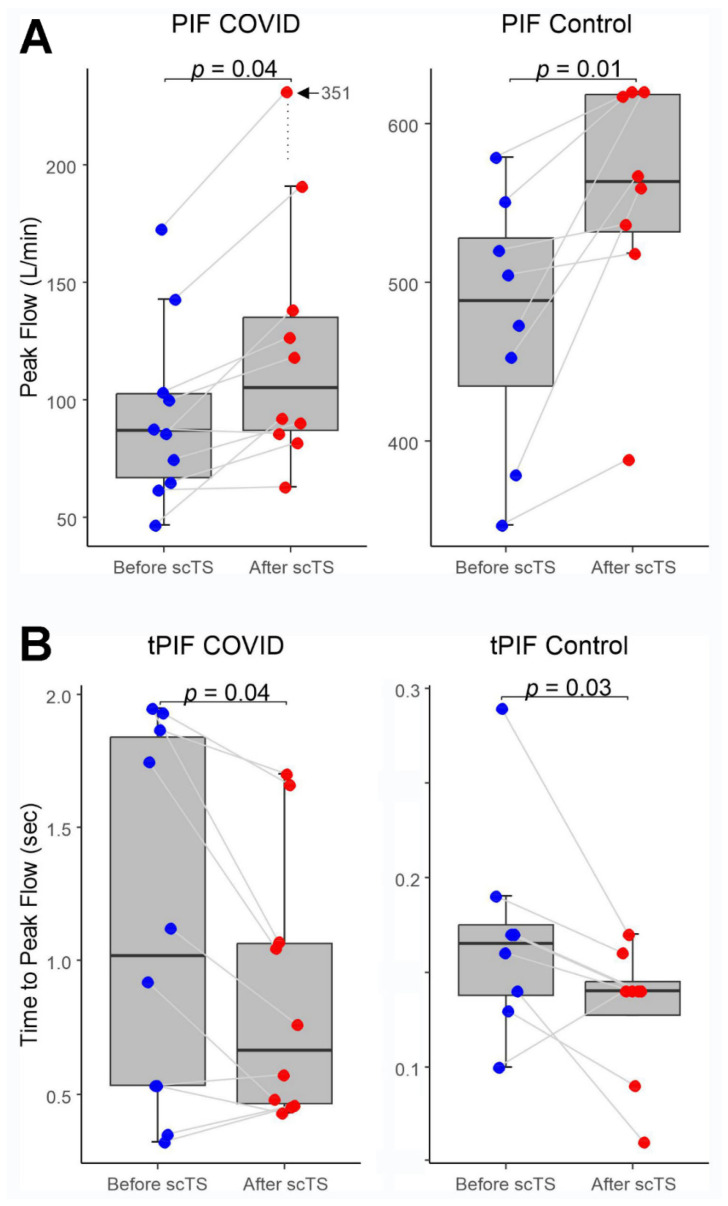
(**A**) Peak inspiratory flow (PIF) and (**B**) Time-to-peak inspiratory flow (tPIF) before and after scTS sessions in post-acute COVID-19 individuals (n = 10) and healthy controls (n = 8). Note different scales representing changes in the COVID and control groups.

**Figure 3:**
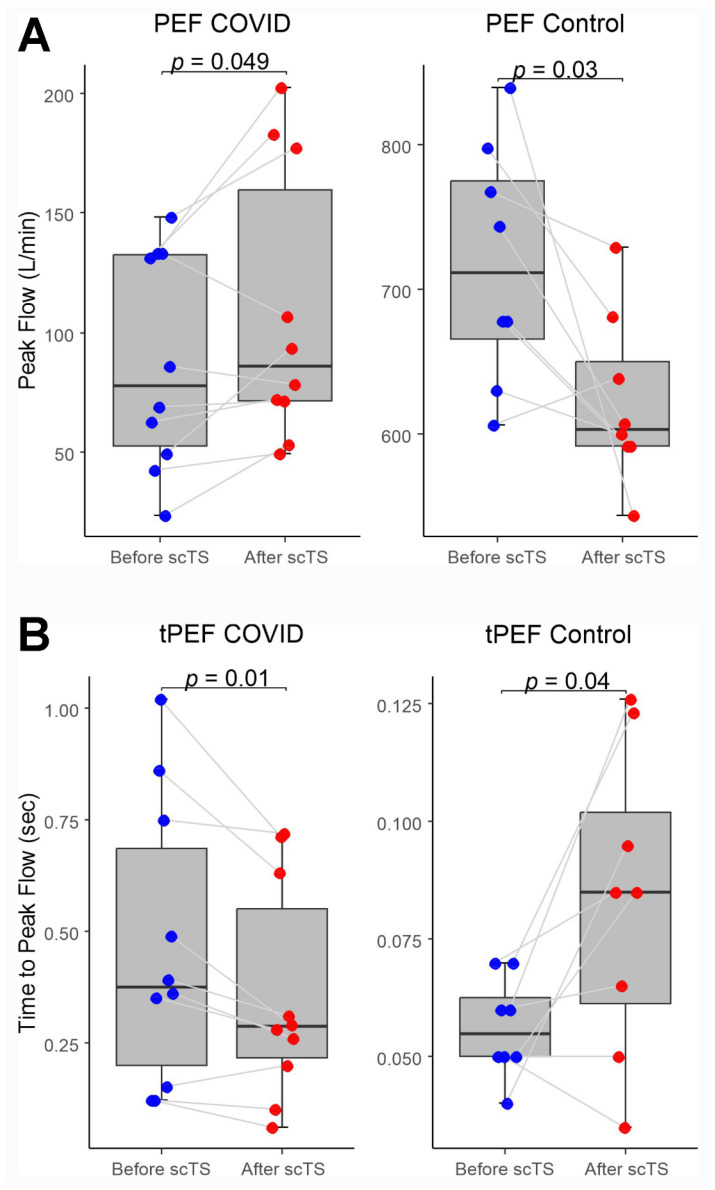
(**A**) Peak expiratory flow (PEF) and (**B**) Time-to-peak expiratory flow (tPEF) before and after scTS sessions in post-acute COVID-19 individuals (n = 10) and healthy controls (n = 8). Note different scales representing changes in the COVID and control groups.

**Figure 4:**
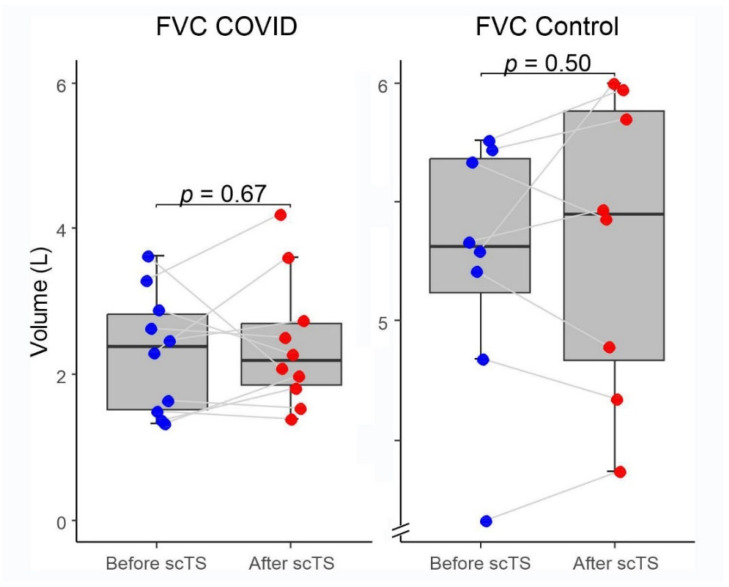
Forced vital capacity (FVC) before and after scTS sessions in post-acute COVID-19 individuals (n = 10) and healthy controls (n = 8). Note different scales representing changes in the COVID and control groups.

In the COVID-19 group, application of scTS led to significantly increased PIF (93.99 ± 38.26 vs. 133.67 ± 84.54 L/min, Mean ± SD; *p* = 0.040) and PEF (87.84 ± 45.05 vs. 108.85 ± 57.31 L/min; *p =* 0.049) in association with significantly decreased tPIF (1.13 ± 0.69 vs. 0.86 ± 0.49 s; *p* = 0.035) and tPEF (0.46 ± 0.32 vs. 0.35 ± 0.24 s; *p* = 0.013). In the control group, exposure to the scTS resulted in significantly increased PIF (475.80 ± 80.79 vs. 553.76 ± 77.76 L/min, *p* = 0.010) and significantly decreased tPIF (0.17 ± 0.06 vs. 0.13 ± 0.04 s; *p* = 0.031). In contrast to the results for the COVID group, there was a significant decrease in PEF (717.75 ± 82.76 vs. 622.76 ± 58.53 L/min; *p* = 0.028) in association with significantly increased tPEF (0.06 ± 0.02 vs. 0.08 ± 0.03 s; *p* = 0.036) (Figure 2 and Figure 3). There were no changes for FVC after scTS in both groups (2.31 ± 0.83 vs. 2.42 ± 0.89 L; *p* = 0.67/COVID group/and 5.25 ± 0.54 vs. 5.33 ± 0.62 L; *p* = 0.503/control group/) (Figure 4).

## 4. Discussion

This is the first publication demonstrating the implementation of scTS as a neuromodulation technique in patients with COVID-19 respiratory deficits. The significant scTS-induced changes in PIF and PEF indicate increased respiratory neuromuscular network activity for both inspiration and expiration in individuals with post-COVID-19 respiratory deficits. In addition, a significant decrease in tPIF and tPEF in these patients indicates improved inter-neuronal activity of the respiratory networks. There were no changes for FVC after scTS in both groups, indicating that pulmonary components were not affected by scTS. This report aimed to present the initial findings regarding the potential of spinal neuromodulation in the post-acute COVID-19 population using a simple “pre-/post-acute effect” study design where baseline data sets served as self-controls in combination with an evaluation of the healthy controls which underwent the same intervention. The results of this study suggest that non-invasive scTS targeting respiratory neuronal networks [28] can be a valuable element of a successful respiratory rehabilitation strategy for these patients.

In the present study, to affect the spinal structures responsible for accessory respiratory motor control [29,30], scTS was delivered using two cathodes placed over the T5 and T10 spinal cord segments and two interconnected anodes positioned at the umbilical level. To exclude respiratory muscle contraction during the intervention, the intensity of scTS was maintained at a sub-motor threshold level. In addition, to exclude the direct effect of scTS, post-intervention testing was performed after a 15-min “wash out” period. In individuals with cervical motor-discomplete injury, we observed that intercostal muscles were activated when the respiratory effort was assisted by the epidural stimulation applied at the lumbar segments [31]. Recently, we demonstrated that a single 20-min session of scTS exposure over the cervical spinal cord resulted in increased excitability of not only spinal but also cortical networks lasting for 75 min after cessation of stimulation [32]. These findings suggest that scTS delivered to T5 and T10 spinal segments may also have a facilitatory effect on supraspinal respiratory structures and further contribute to the increased respiratory activity via descending pathways.

One might expect that scTS could also activate the autonomic network associated with innervation of the airways and subsequently increase lung capacity. However, our results showed that FVC was not altered by stimulation. Our previous work showed that scTS can induce autonomic activation in association with an increase in BP in individuals with spinal cord injury-induced autonomic deficits [19]. In the present study, we did not observe scTS-induced changes in BP. These effects might be linked to the different pathophysiological mechanisms of these two populations and specific parameters of scTS that were used in the two distinct protocols.

This study revealed that in healthy individuals, the scTS affects inspiratory and expiratory motor networks differently. As well as in the COVID-19 group, in the control individuals, we observed activation of inspiratory motor circuitry manifested by significantly increased PIF in association with significantly decreased tPIF. However, there were opposite effects on the expiratory circuitry: a significant decrease in PEF accompanied by significantly increased tPEF indicating suppression of expiratory neuromuscular activity. Observed for the first time, these phenomena suggest that intact and affected spinal networks may employ different regulatory mechanisms in response to the scTS.

The therapeutic potential of non-invasive spinal cord stimulation for patients with respiratory deficits has not yet been studied [23]. Further research is needed to define specific pathophysiological mechanisms related to neural dysfunction in patients with COVID-19, which is a key element for the creation of appropriate rehabilitative strategies for this patient population. It should be noted that respiratory-related data from patients with COVID-19 are currently limited to a single randomized controlled study showing that 6 weeks of general respiratory rehabilitation can improve respiratory function, quality of life, and mental health of elderly patients [33]. A study of respiratory function during clinical recovery and 6 weeks after discharge in COVID-19-induced pneumonia survivors found that although pulmonary function had improved, some restrictive deficits persisted [34]. Based on the results of our previous work [35], we propose that combined with scTS, a specific use-dependent rehabilitative intervention in the form of respiratory training, could result in the enhanced plasticity of respiratory neuronal networks leading to the improved respiratory motor function needed to overcome respiratory deficits developed in individuals with post-acute COVID-19.

### Limitations

This study included unmatched controls with a notable difference in baseline parameters due to substantial respiratory deficits in individuals with post-COVID syndrome and no such deficits in the healthy controls. For the future, full-scale clinical trials; a test-retest approach; and matched control groups, including healthy controls, and/or experimental controls, and/or controls with non-specific scTS configuration, will be needed. In addition, based on our experience of using scTS for locomotor rehabilitation [18,32,36,37], this initial scTS protocol for respiratory neuromodulation can be improved. For example, relocation or addition of the anodes to the anterior upper thoracic region might be more effective due to closer proximity to the cathodes. These questions should be addressed by investigating computational and functional effects of different scTS electrical parameters and electrode positioning.

## 5. Conclusions

A single session of scTS applied over the thoracic spinal cord in post-COVID-19 patients leads to improved respiratory functional performance via activation of the spinal neural networks. These findings indicate that a specifically configurated non-invasive spinal cord stimulation paradigm could be utilized for rehabilitation to reduce persistent respiratory motor deficits in patients recovering from COVID-19.

## Figures and Tables

**Table 1:** Demographic Summary of COVID-19 Participants.

ID	Age (y)	Sex	Height (cm)	Weight (kg)	BMI (kg/m^2^)	FVC (% pred.)
4C	57	M	170	76	26.30	66
5C	47	F	170	62	21.45	70
8C	38	F	168	105	37.20	72
9C	59	M	182	110	33.21	66
10C	34	M	178	88	27.77	71
11C	64	M	170	75	25.95	65
12C	47	M	170	100	34.60	68
13C	76	F	169	71	24.86	65
14C	69	F	156	79	32.46	66
15C	59	F	164	130	48.33	68
Mean ± SD	55 ± 13	NA	170 ± 7	90 ± 21	31 ± 8	68 ± 3

Abbreviations: BMI, body mass index; F, female; FVC, forced vital capacity; ID, identification; M, male; NA, not applicable; pred., predicted.

## Data Availability

The data presented in this study are available on request from the corresponding author.
